# Narrow Range of Coagulation of Ion Associates of Poly(styrene sulfonate) with Alcian Blue Dye

**DOI:** 10.3390/molecules29174017

**Published:** 2024-08-25

**Authors:** Dorota Ziółkowska, Alexander Shyichuk, Iryna Shyychuk

**Affiliations:** Faculty of Chemical Technology and Engineering, Bydgoszcz University of Science and Technology, Seminaryjna 3, 85-326 Bydgoszcz, Poland; szyjczuk@pbs.edu.pl (A.S.);

**Keywords:** ionic association, charge neutralization, cationic dye, anionic polyelectrolyte

## Abstract

The ionic association of Alcian Blue dye with poly(styrene sulfonate) in aqueous solutions was studied for analytical purposes. The quadruple-charged cationic dye, Alcian Blue, was found to form colloidal ionic associates with poly(styrene sulfonate) anions. When the amounts of opposite charges are nearly equal, the resulting ionic associates lose solubility and coagulate rapidly. This effect occurs within a narrow range of the ratio of poly(styrene sulfonate) to Alcian Blue. At the point of charge equivalence, the zeta potential of the resulting particles is zero, which facilitates flocculation. The resulting flocs enlarge to approximately 0.05–0.5 mm and precipitate rapidly. FTIR spectroscopy confirms that the precipitate contains both poly(styrene sulfonate) and Alcian Blue dye. Sedimentation kinetics was studied in detail using scanning turbidimetry. Due to the high molar absorbance of the Alcian Blue dye at 600 nm, the point of equimolar charge ratio was precisely determined by spectrophotometry. The complete precipitation of ionic associates occurs when the amount of poly(styrene sulfonate) ranges from 1.4 to 1.55 mmol per 1 g of Alcian Blue dye. Such a narrow coagulation range allows for the use of the studied effect for quantitative analysis. Both Alcian Blue dye and poly(styrene sulfonate) can be quantified if one of their concentrations is known.

## 1. Introduction

In aqueous solutions, ionic macromolecules create an increased charge density and therefore strongly attract oppositely charged ions. The association of a polyelectrolyte with counterions increases as its molecular weight increases [1]. In the case where the oppositely charged ions are single-charged amphiphiles, electrostatic attraction is accompanied by hydrophobic interactions, often resulting in phase separation. Possible scenarios depend largely on the ratio of amphiphilic to macromolecular charges. When the amounts of opposite charges are equal, the resulting ionic associates usually lose solubility and coagulate [2]. When the charge ratio is far from stoichiometric, the resulting ionic associates have excess charge and may be colloidally stable [2,3].

When counterions have multiple charges, their electrostatic attraction to ionic macromolecules increases proportionally. Therefore, oppositely charged polyelectrolytes form particularly strong complexes [4]. Polyelectrolyte complexes have tunable properties and are therefore an attractive topic in various areas of science. For example, polyelectrolyte complexes have many biomedical [5,6] and environmental [7] applications. Polyelectrolyte complexes can be used in 3D printing techniques [8,9,10]. Polyelectrolyte complexes can also be used to build dielectric layers. Examples are poly(vinylidene fluoride) latex with chitosan [11,12] and polyethyleneimine with poly(acrylic acid) and clay [13]. It was noticed that the best dielectric properties are at the equimolar amounts of oppositely charged polyelectrolytes. For example, poly(vinylidene fluoride)@chitosan dielectric films with equimolar charge ratios have the highest values of breakdown strength and capacitor energy density [11].

The concept of the equimolar charge ratio has proven particularly useful in studying colloidal suspensions of polyelectrolyte complexes. When the charge ratio is close to being equimolar, the properties of colloidal suspensions change abruptly. The greatest changes occur in the charge of colloidal particles, which reverses at the equimolar point [11,14]. At the charge neutralization point, the hydrodynamic radii of polyelectrolyte complexes increase significantly [14]. Near the equimolar ratio, the suspensions have minimal surface tension, while the supernatants have maximal surface tension [14]. The spectacular macroscopic phenomenon is the loss of colloidal stability of polyelectrolyte complexes [11,14]. The colloidal stability of equimolar complexes substantially depends on the properties of the constituent polyelectrolytes. A weak polyelectrolyte complex is a rather soft coacervate equilibrated with polyions in a solution, whereas a strong polyelectrolyte complex forms a solid-like precipitate, resulting in complete phase separation [14].

**Figure 1:**
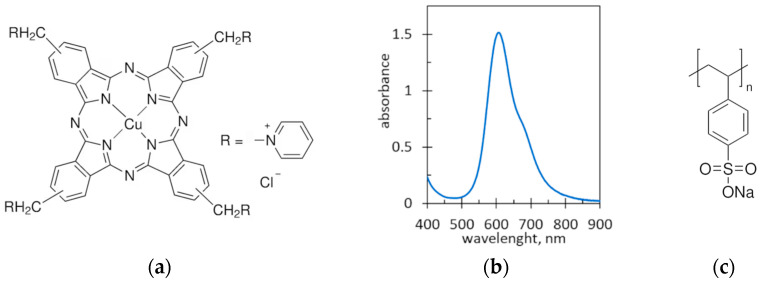
(**a**) Alcian Blue-tetrakis(methylpyridinium) chloride; (**b**) the absorption spectrum of Alcian Blue dye solution of 47.9 mg/L; (**c**) Poly(sodium 4-styrenesulfonate).

The formation of strong ion associates can be used to measure the charge density of polyelectrolytes. For example, surface tension measurements allow for the exact determination of the charge stoichiometry [14]. The fast coagulation of polyelectrolyte colloids was used for the quantitative determination of carrageenan using turbidimetric titration with poly(diallyldimethyl ammonium) chloride [15]. In this context, ion associates of polyelectrolytes with oppositely charged dyes have the advantage that coagulation measurements can be easily performed by spectrophotometry. However, typical dyes are mono-charged and therefore produce weak ion associates that are sensitive to changes in pH and ionic strength. To improve the electrostatic attraction, the quadruple-charged cationic dye Alcian Blue (AB) was used in the present study (Figure 1a). Fortunately, AB dye has quite a high molar absorbance, which facilitates spectrophotometric measurements. AB absorbs light in the wavelength range from 550 to 750 nm, with a maximum of around 605 nm (Figure 1b). In this article, the interaction of AB dye with poly(styrene sulfonate) (Figure 1c) was studied. Poly(styrene sulfonate) (PSS) is known to form strong ion associates with cationic surfactants [16], cationic polymers and cationic dyes [17,18]. PSS ionic associates are prone to further agglomeration due to the presence of multiple aromatic rings in PSS macromolecules, which interact with each other via π–π stacking. As a result, the ionic associates of PSS rapidly undergo phase separation.

No comprehensive reports on the interaction of PSS and AB have been published so far. The only report briefly describes the interaction of AB with PSS among other anionic polymers [19]. Therefore, this paper describes the ion association of PSS and AB, with particular emphasis on aggregation and sedimentation.

## 2. Results and Discussion

### 2.1. Coagulation of Ion Associates of Poly(styrene sulfonate) with Alcian Blue Dye

**Figure 2:**
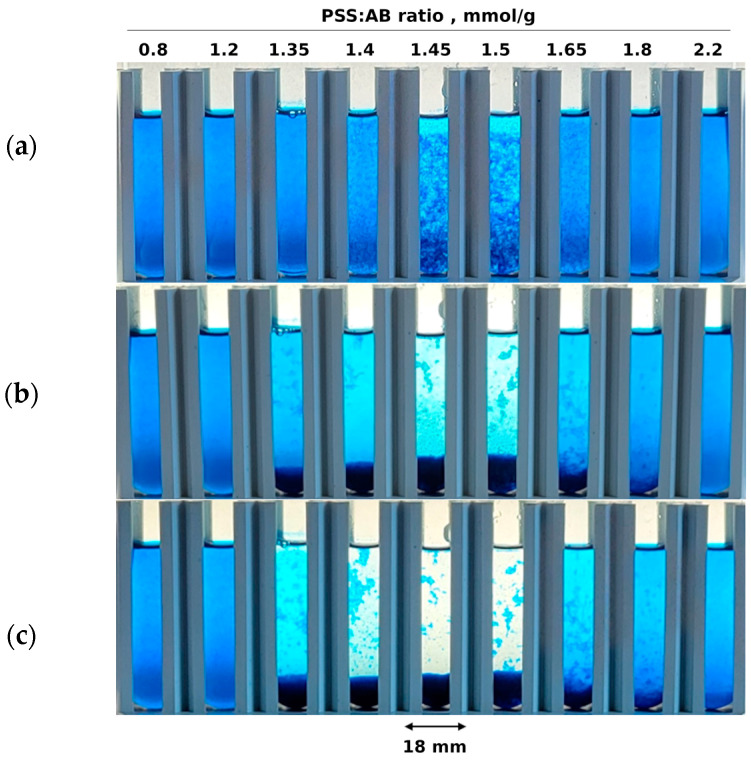
Images of PSS-AB mixed solutions taken after (**a**) 5 min, (**b**) 1 h and (**c**) 24 h of sedimentation. The PSS:AB ratios are the same for the corresponding samples in parts a, b and c of the figure. The concentration of AB dye is 275 mg/L in all of the samples. The sample volume is 7 mL.

As shown in Figure 2, coagulation occurs in a rather narrow range of PSS doses. At the end of the coagulation window, PSS doses vary from 1.35 to 1.65 mmol/g. However, in the case of oppositely charged polyelectrolytes, the coagulation window is broader. For example, intense coagulation of PSS and poly(diallyldimethylammonium chloride) occurs at a molar charge ratio of 0.6 to 1.7 [14]. This wide range of coagulation is likely due to the cooperative aggregation of entangled macromolecules because these polyelectrolytes interact strongly. For comparison, the weak polyelectrolyte poly(acrylate sodium) and the strong polyelectrolyte poly(diallyldimethylammonium chloride) coagulate in the narrower range of a molar charge ratio of 1 to 1.7 [14]. Another example is the coagulation of poly(vinylidene fluoride) latex with carboxylate anions by chitosan, which occurs at the mass fraction of chitosan from 0.4 to 0.9% [11]. Against this background, the narrow coagulation window in the PSS-AB system is a promising premise for studying this phenomenon in the context of using it in quantitative analysis.

### 2.2. Particle Size and Charge

**Figure 3:**
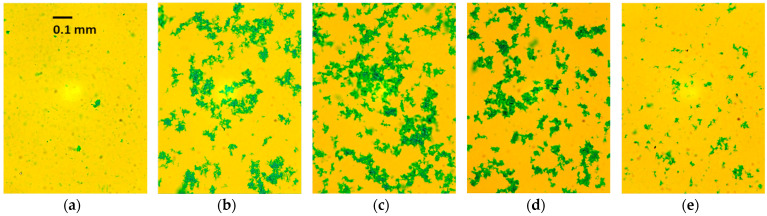
Micro-images of the precipitates obtained at PSS/dye ratios of (**a**) 1.2; (**b**) 1.35; (**c**) 1.45; (**d**) 1.65; and (**e**) 1.9 mmol/g. The AB concentration is 38.4 mg/L.

Micro-images of the suspensions containing a constant dose of AB dye and various doses of PSS illustrate the coagulation process (Figure 3). Both samples with a molar excess of AB (Figure 3a) or PSS (Figure 3e) contain fine particles (up to approx. 50 µm). Such small particles remain suspended for a long time (compare Figure 2c). Within a narrow range of component proportions, the particles of ion associates stick together (Figure 3b–d), forming large flocs with a size of approx. 0.3 mm and larger. This causes rapid sedimentation (Figure 2).

**Figure 4:**
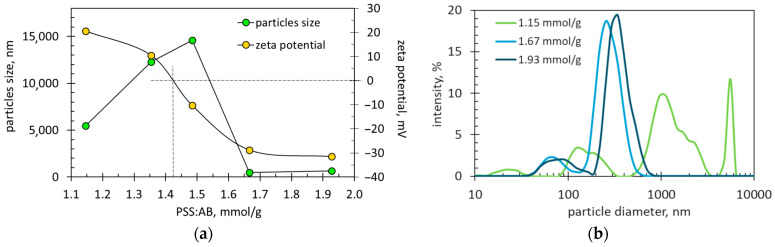
(**a**) The average size and zeta potential of suspended particles depending on the ratio of PSS amount to AB mass. The dotted lines indicate the point of zero potential. (**b**) Particle size distributions in suspensions with indicated PSS:AB ratios. The dye concentration in all samples is 287.6 mg/L.

Particle size and charge measurements using a Zetasizer confirmed significant changes depending on the PSS-to-AB ratio (Figure 4). At a low PSS-to-AB ratio of 1.15–1.35 mmol/g, the particles of ionic associates carry a positive charge (Figure 4a). The identical charge of suspended particles prevents their aggregation (compare Figure 3) and ensures colloidal stability (compare Figure 2). An increase in the PSS-to-AB ratio to 1.49 mmol/g and above causes the charge of the suspended particles to change from positive to negative (Figure 4a). Under the conditions of a uniform negative charge, the dispersed particles also show stability, as illustrated in Figure 2 and Figure 3.

It is worth noting that the negative zeta potential reaches higher absolute values (i.e., 29–32 mV) compared to those of positive zeta potential (20.6 mV). The reason is that the negative charge comes from the excess of PSS, while the positive charge comes from the excess of AB. PSS is well known as a strong anionic polyelectrolyte due to its well-dissociated sulfonate groups [20]. The actual dissociation degree is difficult to determine due to the strong attraction between polyions and counterions. This effect, known as counterion condensation, reduces the effective charge of polyelectrolytes and polyelectrolyte complexes [21].

Using interpolation of the experimental relationship of zeta potential, the critical ratio of PSS to AB was found to be 1.424 mmol/g (Figure 4a). It is the ratio of PSS to AB that provides equimolar amounts of negative and positive charges. The obtained value agrees well with the coagulation window determined on the basis of sedimentation tests (Figure 2c). The zero charge on the particles allows them to flocculate, which causes the particle size to increase. Therefore, the size of suspended particles reaches a maximum near the critical PSS-to-AB ratio (Figure 4a). This relationship agrees well with the microscopic observations shown in Figure 3. Very similar relationships of zeta potential and particle size with the component ratio were observed in the mixtures of poly(vinylidene fluoride) with chitosan [11]. With the increase in the amount of the cationic component (chitosan), the charge of the suspended particles changes from negative to positive, whereas the particle size increases rapidly and reaches a maximum only at zero particle charge [11]. This fact clearly indicates that it is the zero charge that causes the aggregation of particles.

The numerical values of the particle sizes in Figure 4a are different from the particle sizes recorded in the micro-images (Figure 3). This is because extensive flocculation occurs in the tested suspensions. Figure 3 shows quite large flocs, while small particles are also present in the suspensions. Particles smaller than 1 μm are invisible in the microscopic image; however, Figure 4b reveals their presence. The particle size distributions in Figure 4b are multimodal, indicating that flocculation is ongoing. It is likely that the flocs’ equilibrium state was disturbed during the measurement.

### 2.3. Composition of the Ionic Associates

**Figure 5:**
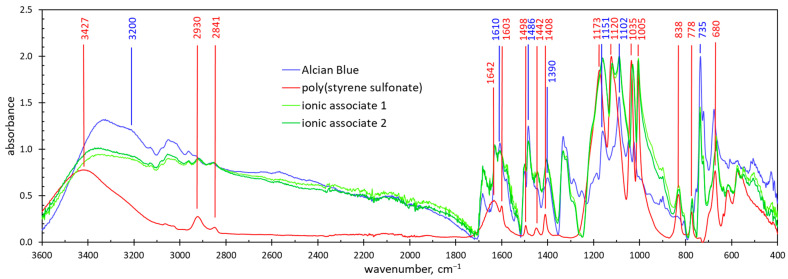
The FTIR spectra of poly(styrene sulfonate), Alcian Blue dye and their ionic associates at the equimolar PSS:AB ratio. The concentration of the AB dye solution used to form the associates was 767 mg/L (associate 1) and 1150 mg/L (associate 2).

Figure 5 shows the FTIR spectra of ionic associates precipitated at the equimolar PSS:AB ratio. The spectra contain the bands characteristic of both poly(styrene sulfonate) [22] and Alcian Blue dye [23].

The absorption bands at 1603, 1498, 1408, 1120 and 1005 cm^−1^ are characteristic of the PSS aromatic ring [22,24,25]. The sharp peak at 1005 cm^−1^ is attributed to the in-plane bending vibration of the benzene ring [1,26]. The bands at 1442 cm^−1^ and 838 cm^−1^ indicate a benzene ring disubstituted in the para positions [25]. The peaks at 778 and 680 cm^−1^ are attributed to the C–H vibration in the aromatic ring [25,27]. The absorption bands at 2910 and 2843 cm^−1^ are attributed to the stretching vibration of the –CH_2_– group [24,25].

The presence of sulfonate anions is manifested by strong absorbance bands at 1035 and 1173 cm^−1^, which are attributed to the symmetric and antisymmetric stretching of the O=S=O group, respectively [22,24,25,26]. The asymmetric and symmetric vibrations of the sulfonate group also result in the bands at 1498 cm^−1^ and 1408 cm^−1^ [27]. These bands can also be ascribed to the vibration of the aromatic ring [18]. The broad band centered at 3426 cm^−1^ and the weak band at 1632 cm^−1^ indicate the presence of H_2_O molecules [25,26], probably adsorbed at polar sulfonate groups.

The FTIR spectrum of Alcian Blue dye contains a wide absorption band in the range of 3400 to 3200 cm^−1^ (Figure 5). This band is not assigned to a functional group but is characteristic of Alcian Blue dye [23]. The sharp peaks at 1610, 1486, 1390, 1151, 1102 and 735 cm^−1^ can be attributed to N-substituted pyridinium [28].

The spectra of the precipitated ionic associates contain all of the mentioned peaks (Figure 5). This confirms that the ionic associates contain both poly(styrene sulfonate) and Alcian Blue dye. The spectra of ionic associates obtained at different initial concentrations of AB are almost identical (Figure 5). This fact indicates that the composition of the ionic associates is stable.

### 2.4. Sedimentation of Dye–Polymer Associates

**Figure 6:**
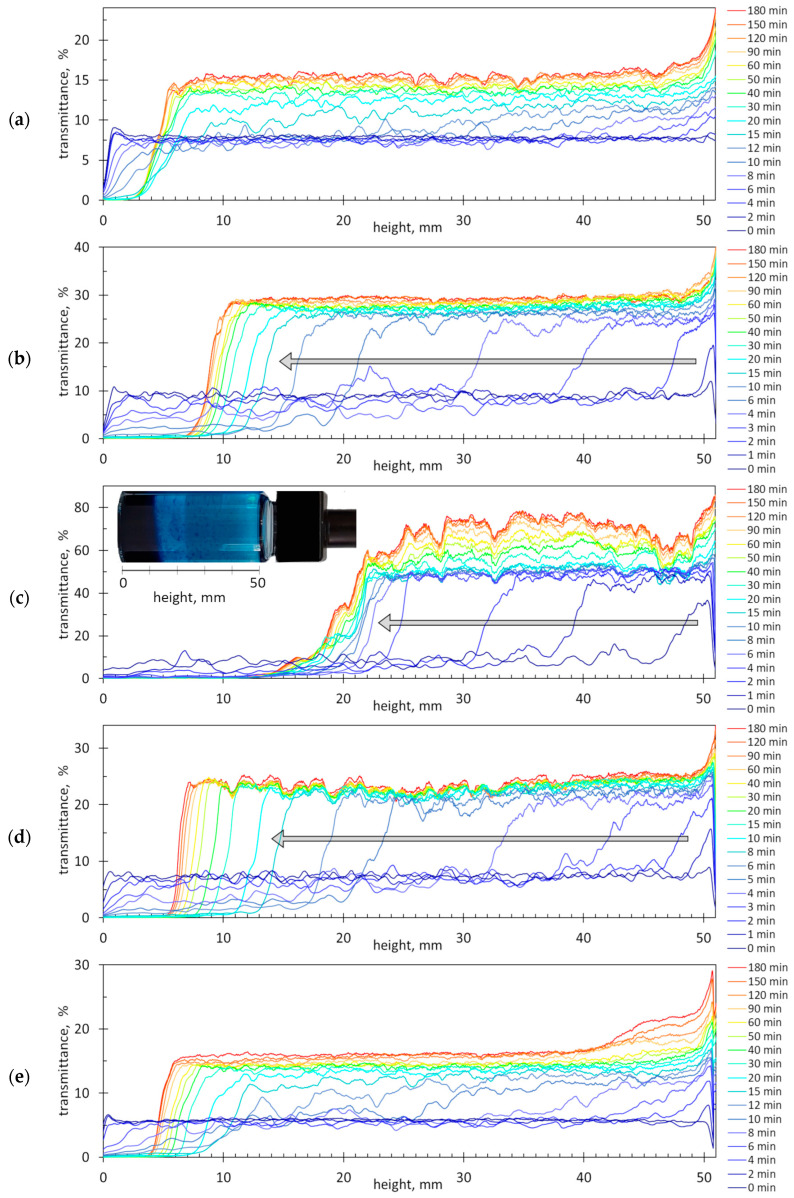
The light transmission profiles during the sedimentation of the mixed polymer–dye suspensions (the most intense sedimentation is indicated by arrows). The dye concentration in all samples is 661.1 mg/L, and the amount ratio of PSS to AB is (**a**) 1.15 mmol/g; (**b**) 1.27 mmol/g; (**c**) 1.47 mmol/g; (**d**) 1.72 mmol/g; and (**e**) 2.80 mmol/g. The sample volume is 26.1 mL.

Figure 6 shows the graphs of light transmittance as a function of the height of the cuvette, recorded successively at an interval of 1 min for mixtures with different PSS:AB ratios. The obtained sedimentation profiles are quite different, indicating that the tested suspensions have different stabilities.

The least stable is the suspension with the PSS:AB ratio of 1.47 mmol/g (Figure 6c). Compared to the other tested suspensions, the initial transmittance is comparable (below 10%), but the final transmittance is the highest (over 70%). This suspension shows the fastest changes in the transmittance profile. The light transmission in the top layer (from 46 to 50 mm) significantly decreases within the first 2 min. The clear top layer expands rapidly within 8–10 min. The random fluctuations on the sedimentation profiles are from large settling flocs (compare Figure 3c), whereas immobile sharp peaks are from small flocs attached to the cuvette walls. After 8 min, an opaque bottom layer of 20–22 mm is formed. The sediment layer is clearly visible in the inserted image of the measuring vessel (Figure 6c). Complete sedimentation takes more than 3 h.

When the PSS:AB ratio differs from the equimolar value of 1.47 mmol/g, sedimentation occurs more slowly, and the formation of the sediment layer takes longer (Figure 6a,b,d,e). Compared with the equimolar suspension, the suspensions with the PSS:AB ratios of 1.27 and 1.72 mmol/g have a lower final transmittance and a lower final height of the sediment layer (Figure 6b,d). The suspension with the PSS:AB ratio of 1.15 mmol/g is rather stable—the transmittance value increases insignificantly, i.e., from 7 to 15%, and the sediment layer is only 5 mm (Figure 6a). The suspension in which the ratio of the components is 2.8 mmol/g behaves similarly (Figure 6e).

**Figure 7:**
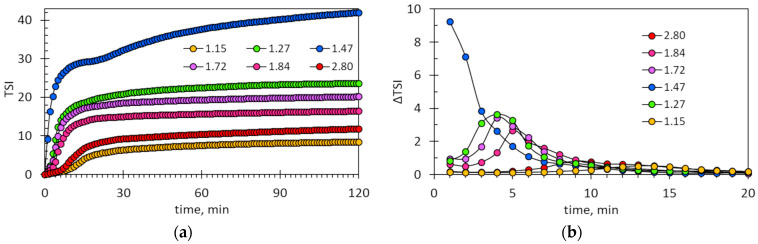
(**a**) Changes in the Turbiscan Stability Index over time. (**b**) The derivatives of the TSI plots. The legends show the values of the PSS:AB ratios expressed in mmol/g.

Figure 7a shows the values of the Turbiscan Stability Index (TSI). The parameter TSI indicates relative changes in the tested suspensions. Three stages are visible in the TSI graphs: an initial slow increase in TSI values, and then a rapid increase followed by a final slow increase. These stages are more clearly visible in Figure 7b, which shows the time derivatives of the TSI function (ΔTSI). The first step is the induction time required for the agglomeration of hydrophobic ionic associates. The derivative TSI plot at the equimolar PSS:AB ratio does not show any induction time, which indicates that quick agglomeration occurs. The second stage is characterized by rapid changes in TSI values and corresponds to the rapid sedimentation of large flocs. The shift in the position of the maxima, which determine the point of inflection of the curves, well reflects the relationship between the induction time and the ratio of the mixture components, too. The final step, with minor changes to the TSI values, is the slow sedimentation of the remaining fine particles. The fine particles are well suspended and require quite a long time to settle (Figure 7a).

**Figure 8:**
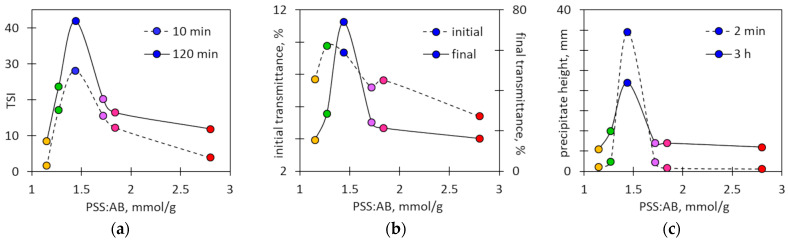
The characteristic sedimentation parameters read from Figure 6 and Figure 7. (**a**) The values of the TSI after 10 min and 120 min of sedimentation; (**b**) the initial and final transmittances at the height of 30 mm; and (**c**) the initial and final height of the precipitate depending on the PSS:AB ratio. The colors of the points refer to the PSS:AB ratio values and are the same as in Figure 7.

The values of the TSI at different PSS:AB ratios clearly indicate the maximum at the ratio of 1.47 mmol/g (Figure 8a). The same inference comes from the graphs of the final suspension transmittance (Figure 8b) and the height of the precipitate (Figure 8c). It is worth noting that only with a nearly equimolar composition of the mixture are the agglomerates formed large enough for the precipitate to be visible in the entire sample volume in the first minutes (compare Figure 2a and Figure 8c). Thus, Figure 6, Figure 7 and Figure 8 clearly indicate that the rapid sedimentation of ionic associates of PSS and AB takes place in the narrow range of the PSS:AB ratio.

### 2.5. Determination of Charge Equivalence Point by Photometric Measurements

**Figure 9:**
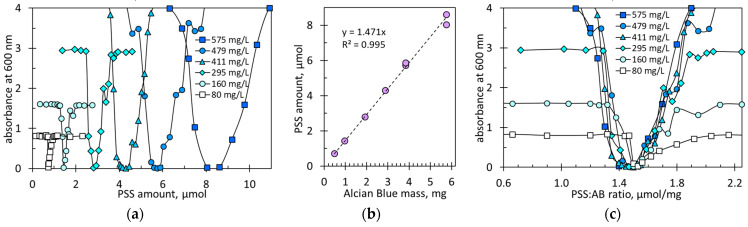
(**a**) Absorbance values in mixed polymer–dye solutions after 24 h of sedimentation. AB concentration in samples is indicated. (**b**) Critical PSS amount vs. AB mass in solution. (**c**) Absorbance of solutions with indicated AB concentrations depending on ratio of PSS amount to AB mass.

Figure 9a shows the absorbance of the PSS-AB mixtures, obtained using dye solutions with various concentrations, after sedimentation for 24 h. In certain ranges of added amounts of PSS, the absorbance values are strongly reduced. This indicates that the AB dye has been incorporated into the ionic associate and coagulated. The recorded U-shaped absorbance relationship agrees well with the bell-shaped transmittance relationship in Figure 8b. The coagulation range is widened with increasing AB concentration. However, complete sedimentation only occurs when absorbance values are zero. This is most likely the point of charge neutralization.

The zero absorbance is observed at certain PSS amounts (Figure 9a). The higher the AB concentration, the more PSS is needed to cause complete sedimentation. Figure 9b shows that the critical amount of PSS at the point of complete sedimentation is directly proportional to the quantity of AB in the solution. In other words, the critical ratio of the PSS amount to the AB amount is a constant value. The numerical value of the charge equivalence ratio was found to be 1.47 mmol/g (Figure 9b). This value is quite consistent with the result of the zetametric measurements (Figure 4a). On the other hand, considering the AB dye’s molecular weight of 1086.4 g/mol and a fourfold charge, the theoretical charge amount per gram should be 3.67 mmol/g. These numbers indicate that the real AB dye content in the commercial AB reagent is approx. 40%. This value differs from the supplier’s declaration that the dye content exceeds 85%. Many commercially available samples of Alcian Blue are known to contain 50% or less dye due to the presence of a stabilizer (boric acid) and fillers (sodium sulfate, dextrin, etc.) [29]. Additionally, long storage with an insufficient stabilizer may result in reduced solubility [30]. Figure 9c shows the same data as in Figure 9a but plotted as a function of the PSS:AB ratio. The presented plots indicate that complete coagulation occurs in the range of a PSS:AB ratio from 1.4 to 1.55 mmol/g. Thus, absorbance measurements allow for a fairly accurate determination of the charge equivalence point.

**Figure 10:**
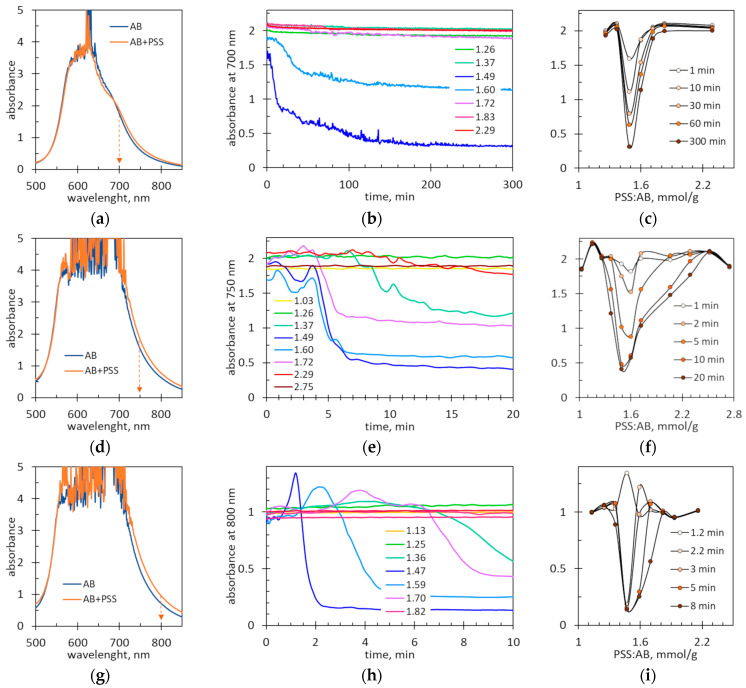
(**a**,**d**,**g**) The spectra of the AB dye and equimolar AB-PSS mixtures. The arrows show the wavelengths for recording the kinetic lines. (**b**,**e**,**h**) The absorbance changes over time in the mixed polymer–dye suspensions at the indicated values of the PSS:AB ratio expressed in mmol/g. (**c**,**f**,**i**) Absorbance as a function of the PSS:AB ratio at indicated times. The AB concentration in the samples is (**a**–**c**) 167 mg/L, (**d**–**f**) 500 mg/L and (**g**–**i**) 834 mg/L.

The spectrophotometric measurements were performed taking in mind the possible application of the coagulation phenomenon for analytical purposes. However, the long analysis time (e.g., 24 h, as in Figure 9) would be a disadvantage of the method. Therefore, increased concentrations of AB were tested. The absorbance at the main peak of the AB dye was too high, so measurements were performed at longer wavelengths. Figure 10a,d,g show the spectra of the AB dye and equimolar AB-PSS mixtures. The corresponding spectra of the dye and the dye–polymer mixtures are almost identical. This means that the complexation phenomenon does not affect the optical properties of AB. The long wavelength tail of the AB spectrum is decreasing. This allows for the selection of the working wavelength for samples with a high dye concentration. This way is more convenient than using measuring cuvettes with a short optical path. The working wavelengths were selected so that the maximum absorbance values were within the range of 0.9–2.1.

Figure 10b,e,h show the changes in the absorbance values over time in samples with different AB concentrations. The initial parts of the recorded kinetic lines depend on the dye concentration. When the dye concentration is low, small absorbance oscillations are observed coming from small suspended particles (Figure 10b). As the dye concentration increases (Figure 10e,h), visible maxima appear on the lines. They are particularly visible with a large amount of precipitate, i.e., for a PSS:AB ratio of 1.37–1.72 mmol/g at a moderate dye concentration (Figure 10e) and for a PSS:AB ratio of 1.36–1.70 mmol/g at a high dye concentration (Figure 10h). The maxima are related to the momentary increase in the number of sediment particles in the measuring window as the sediment settles along the cuvette. With the largest amount of sediment, the maximum absorbance value significantly exceeds the initial absorbance value (Figure 10h). The multiple peaks visible in the sedimentation curves (Figure 10e) are probably due to the fact that flocs of different sizes settle at different rates.

Regardless of the shape of the initial part of the kinetic line, it can be easily seen that some suspensions settle rapidly, whereas other suspensions are quite stable. As expected, the least stable suspensions are those with the equimolar charge ratio, i.e., near to 1.47 mmol/g. Figure 10c,f,i show the absorbance values at selected time intervals depending on the PSS:AB ratio. Figure 10c,f,i confirm that the equimolar charge ratio lies between 1.45 and 1.5 mmol/g.

Another important conclusion from the comparison of kinetic curves is that the sedimentation rate increases with increasing dye concentration. At low AB concentrations, almost complete sedimentation of the suspension at an equimolar charge ratio takes 200–300 min (Figure 10b). At higher AB concentrations, the sedimentation time is shortened to 3–8 min (Figure 10e,h). The possibility of shortening the test time by selecting reagent concentrations is an undoubted advantage of spectrophotometric measurements.

## 3. Materials and Methods

### 3.1. Materials

Alcian Blue-tetrakis(methylpyridinium) chloride came from Sigma-Aldrich (Burlington, MA, USA). According to the supplier, the dye content is equal to or greater than 85%. The aqueous AB stock solution was filtered and stored in the dark. Working solutions of various concentrations were obtained by diluting the stock solution with distilled water.

Poly(sodium 4-styrenesulfonate) came from Sigma-Aldrich. According to the supplier, the average molecular weight of PSS is approximately 70,000 g/mol. An aqueous PSS stock solution with a concentration of 2.87 mM was used.

### 3.2. Methods

Suspensions of PSS-AB ionic associates were obtained by adding a dose of PSS stock solution to a fixed dose of AB solution of a specific concentration and supplementing with distilled water to the same volume in a given measurement series.

Micro-images in polarized light were taken approximately 0.5 h after sample preparation using a B-500 optical microscope (Optika, Ponteranica, Italy). The charge and size of suspended particles were determined for mixtures stabilized for at least 24 h by Laser Doppler Electrophoresis and Dynamic Light Scattering, respectively, using Zetasizer Nano-ZS (Malvern Panalytical, Malvern, UK). ATR-FTIR spectra were recorded using an Alpha-P spectrometer (Bruker, Billerica, MA, USA) with a diamond window.

Sedimentation profiles were recorded with a Turbiscan LAB scanning turbidimeter (Formulaction, Toulouse, France) operating at 880 nm in transmittance mode. The Turbiscan Stability Index (TSI) was calculated with the following formula:

$${TSI}_{t}=\left(\sum_{h=1}^{H}\left|{transmittance}_{0}(h)-{transmittance}_{t}(h)\right|\right)/H$$

where TSI_t_ is the index value at time t, transmittance_0_(h) is the initial transmittance at height h, transmittance_t_(h) is the transmittance at time t and at height h, and H is the total number of heights under measurement.

Photometric measurements were carried out with a Genesys 50 UV-Vis spectrophotometer (Thermo Fisher Scientific, Waltham, MA, USA) at wavelengths of 600, 700, 750 and 800 nm using glass test tubes with a diameter of 14 mm and disposable cuvettes with an optical path of 4 mm.

## 4. Conclusions

Poly(styrene sulfonate) anions and Alcian Blue dye cations form strong ionic associates. The colloidal stability of the resulting suspension depends on the ratio of PSS to AB. When the amounts of charges of opposite signs are not equal, the resulting suspensions are quite stable. When the opposite charges are equal, the resulting ionic associates lose charge and coagulate abruptly. This effect occurs within a narrow range of the PSS:AB ratio. The fastest coagulation occurs when the amount of poly(styrene sulfonate) ranges from 1.4 to 1.55 mmol per 1 g of Alcian Blue dye. At this PSS:AB ratio, the largest amount of precipitate is formed, and the supernatant becomes almost completely discolored. The concentrations of PSS and AB do not affect the composition of the precipitated ionic associate. The applicability of the presented results is due to the narrow range of coagulation. The clearly visible coagulation of PSS-AB ionic associates is an obvious indicator that the molar amounts of PSS and AB are equal. A narrow coagulation range allows for the determination of PSS or AB quantity by using a simple titration procedure. For this purpose, the AB solution of unknown concentration should be titrated with a standardized PSS solution until coagulation occurs. Conversely, PSS solutions of unknown concentration can be titrated with a standardized AB solution.

## Data Availability

All data are contained within the article.
